# Outcomes of total hip arthroplasty using dual mobility cups following failed internal fixation of proximal femoral fractures at a mean follow-up of 6 years

**DOI:** 10.1051/sicotj/2023038

**Published:** 2024-01-18

**Authors:** Chahine Assi, Joeffroy Otayek, Jad Mansour, Jimmy Daher, Jacques Caton, Camille Samaha, Kaissar Yammine

**Affiliations:** 1 Department of Orthopedic Surgery, Lebanese American University Medical Center-Rizk Hospital, Lebanese American University School of Medicine Beirut Lebanon; 2 Center of Evidence-based Anatomy, Sports & Orthopedic Research Beirut Lebanon; 3 Institut de Chirurgie Orthopédique Lyon France

**Keywords:** Proximal femoral fracture, Femoral neck fracture, Total hip arthroplasty, Dual mobility cup

## Abstract

*Introduction*: Performing total hip arthroplasty (THA) after failed internal fixation of proximal femoral fractures (PFF) is known to be associated with high rates of complications. Dual mobility cups (DMC) are known to lower dislocation events in high-risk patients. Very few reports investigated the outcomes of THA using DMC following failure of internal fixation for PFF. *Methods*: This is a retrospective monocentric continuous study of 31 patients who underwent THA with DMC after failed internal fixation of PFF. The clinical assessment was based on the modified Harris hip score (mHHS) at the last follow-up. The complication rates and radiological analyses were recorded. *Results*: The mean follow-up period was 5.96 ± 4.2 years. At the last follow-up, the mean mHHS was 92.9 ± 9.1 with 71% of the patients describing their operated hip as a forgotten hip. No dislocation or aseptic loosening events were noted. One patient developed a septic loosening of the implant. No significant radiological changes were recorded. Sixteen stems (51.6%) were placed in a neutral position, 13 (42%) in valgus (2.74 ± 1.72°), and 2 (6.4%) in varus (6.94 ± 2.02°). *Conclusion*: This study emphasizes the advantage of using DMC following failed internal fixation of PFF in reducing dislocation and complication events in this high-risk population.

## Introduction

Failure of internal fixation in proximal femoral fracture (PFF) is a common complication ranging from 20% to 30% [1], usually requiring revision by total hip arthroplasty (THA) [2]. However, this procedure can be technically challenging due to the need to remove the hardware, the modified anatomical landmarks, and the potential occurrence of malunion, non-union, and peri-fracture ossification. Sarcopenia and fatty involution related to age and repeated procedures increase the risk of post-operative instability [3, 4]. All these elements can lead to errors in implant positioning putting the hip at higher risk of dislocation especially in extracapsular fractures [5–7]. This is particularly true with failure of extracapsular PFF fixation, which incurs a higher risk of complications than intracapsular fractures (25 vs. 0%, *p* < 0.0001): periprosthetic fracture (9.4%), dislocation (6.3%), surgical-site infection (6.3%), and stem penetration (3.1%) [8]. The same authors observed poorer results for salvage THA with standard cups when compared to dual mobility cups (DMC), notably in terms of hip stability [8].

DMC were designed as a solution to reduce THA dislocation events [9, 10]. Since its introduction in France by Gilles Bousquet in 1974 [11], dual mobility cups (DMCs) have gained significant attention and have been extensively studied in various surgical settings, including trauma, primary, and revision surgeries [12–16]. The DMC design is associated with lower dislocation and re-operation rates in elective surgery and for high-risk patients [17–23]. Recent literature showed excellent survival, radiological, and clinical results of DMC at a mid- and long-term follow-ups [24, 25]. Although research investigating the application of DMCs after failed internal fixation of proximal femoral fractures is limited, a few studies have explored the effect of DMCs on preventing implant dislocation in this specific context [7, 26, 27].

We hypothesized that DMC has low rates of instability when used after failure of PFF along with good functional outcomes. The main objective was to evaluate dislocation and re-operation rates after THA using DMC following failed internal fixation of proximal femur fractures.

## Material and methods

### Study population

This retrospective monocentric study involved the prospective collection of data from patients hospitalized between January 2005 and December 2020. We retrieved and analyzed the medical records of patients who were hospitalized between January 2005 and December 2020 and underwent THA with a contemporary uncemented DMC and a cemented femoral stem following the failure of PFF. Ethical approval was obtained from our institution’s Ethical Committee prior to its commencement. Clinical and radiological evaluations were conducted by an independent observer.

The types of PFF were extracapsular (intertrochanteric and subtrochanteric) and intracapsular (femoral neck fractures, FNF) treated with reduction and insertion of intramedullary nailing, dynamic hip screw, or cannulated screws.

The etiology of failure necessitating conversion of the initial fixation to THA included osteonecrosis of the femoral head, early failure of fixation, and non-union.

The inclusion criteria were set as follows: (a) THA in patients initially treated by internal fixation, (b) the exclusive use of contemporary DMC, (c) the use of a posterolateral approach, and (d) a minimum follow-up of 2 years.

Exclusion criteria were set to be (a) primary THA, (b) the use of standard cups, (c) revision by renewed internal fixation, (d) post-operative follow-up period <2 years, and (e) refusal to consent.

### Surgical technique and implants used

**Figure 1:**
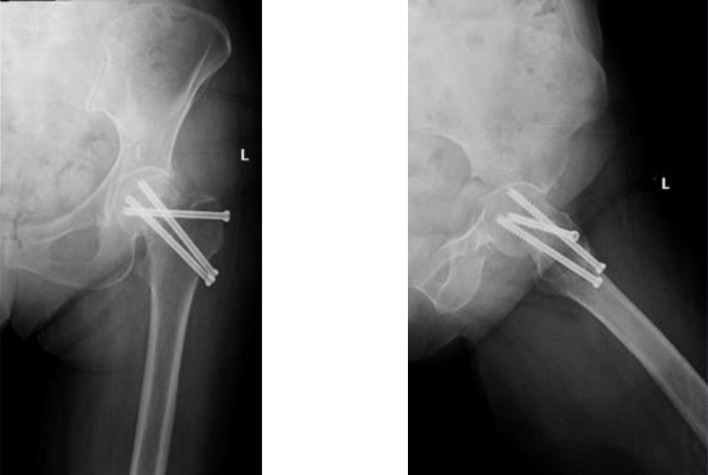
Non-union of FNF following fixation by screws.

**Figure 2:**
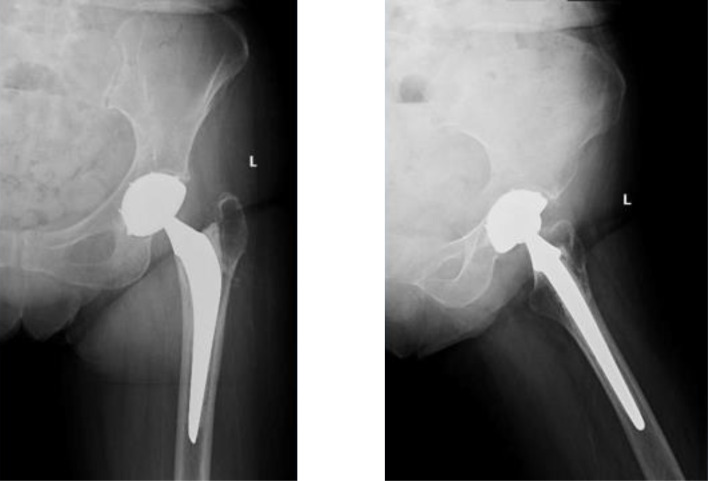
THA with DMC.

**Figure 3:**
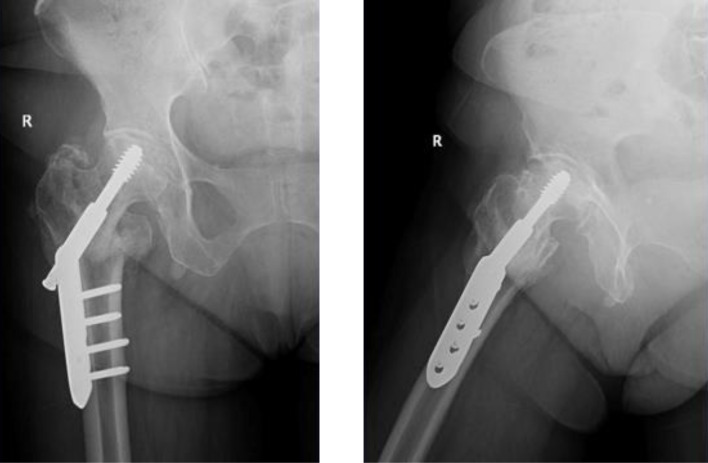
Non-union of FNF following dynamic hip screw.

**Figure 4:**
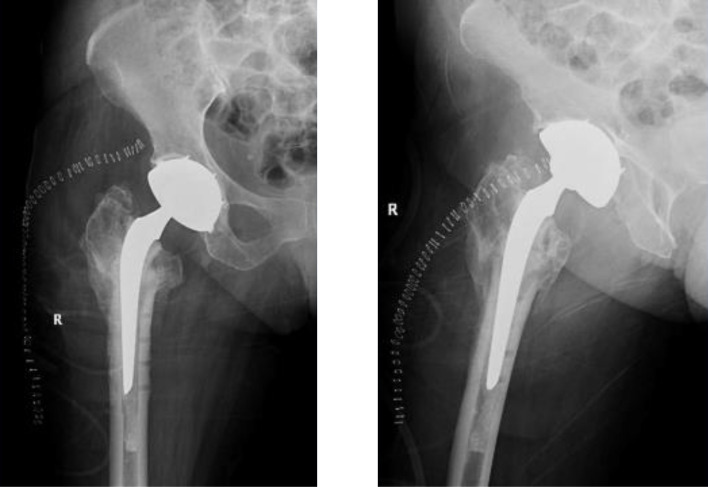
THA using DMC, note the cement plug distal to the last screw.

Patients were positioned in a lateral decubitus position. A posterolateral approach was used in all patients. The hardware removal and the THA were performed in the same setting. The hardware was removed using the same approach and a contemporary uncemented DMC was inserted in all patients. A cemented standard femoral stem (Charnley-Kerboul) was the preferred choice for the procedure. The cement plug was always positioned under the most distal screw hole. In situations where a longer stem was required to span the most distal screw hole, a cementless diaphyseal fixation Targos stem was used (Figures 1–4).

Post-operative rehabilitation protocol with passive and active motion exercises including full weight-bearing ambulation was initiated the next day of surgery.

### Clinical and radiological assessments

Both evaluations were conducted by an independent research orthopedic fellow.

#### Clinical analysis

All complications including infection, aseptic loosening, septic loosening, dislocation, and instability events were thoroughly extracted and recorded. Patients who were unable to travel to the hospital were evaluated by telephone interview for pain, function activity, and the retention of their implants.

The modified Harris hip score (mHHS) [28] was employed to assess the functional status of the operated hip during the final follow-up evaluation. Additionally, patients were queried about whether they perceived their operated hip as a “forgotten hip.” In this context, a “forgotten hip” was defined as the absence of any clinical symptoms or signs, both at rest and during motion.

#### Radiological analysis

Digital standing anteroposterior and lateral views of the pelvis and the operated hip on day 1 after surgery and at last follow-up were used for radiological analysis. In the anteroposterior direction, the central beam was 1.2 m away from the patient and was directed to the midpoint between the upper border of the symphysis and the center between both anterior superior iliac spines. For the lateral radiograph, the focus was centered on the cranial tip of the greater trochanter, with the source situated 1.2 m from the patient. The radiological assessment included the examination for various indicators such as osteolysis, radiolucent lines, signs suggestive of aseptic loosening of the components (such as tilting or migration), and the presence of heterotopic ossification. The stem was categorized as either in varus or valgus alignment if there was a difference of 5° or greater between the stem axis and the anatomical axis of the femur in the coronal plane while ensuring the lower limbs were in a neutral rotation position [26, 27]. Radiolucent lines and osteolysis in the acetabulum were evaluated according to the DeLee and Charnley classification [29]. Periprosthetic femoral radiolucency was assessed using the Gruen and Johnston classification [30, 31].

Patients who died during the follow-up had their clinical and radiological outcomes assessed at their last consultation, provided the follow-up period was more than 2 years.

### Statistical analysis

The StatsDirect (Cambridge, UK) software was used for statistical analysis. Descriptive statistics were fitted to outcome analysis; means were reported with their standard deviations (*SD*). A subgroup analysis was conducted based on the type of the initial fracture: intracapsular versus extra-capsular FNF. To this, the *t*-student test and the chi-square test were used to look for significant differences between means and proportions, respectively. A *p*-value of <0.05 was considered significant.

## Results

### Patient sample demographics

The total sample comprised 31 patients (25 females and 6 males) including 31 hips (14 right and 17 left). The mean age was 71 ± 11 years. None of our patients had a previous lumbar spine surgery. The mean American Society of Anesthesiologists (ASA) score was 2.03 ± 0.60. The initial types of hip fracture were 20 extracapsular with 18 intertrochanteric fractures, and 2 subtrochanteric fractures, and 11 intracapsular FNF. The initial hardware used in the primary surgery was as follows: 17 proximal femoral nails, 11 cannulated screws, and 3 Dynamic Hip System (DHS). The causes of conversion to THA are described in Table 1. The main cause for conversion was secondary hardware displacement. In particular, we had 4 cases of lag screw cut-outs and 2 cases of lag screw cut-through, protruding into the joint line. The mean follow-up period was 5.96 ± 4.2 years, ranging from 2 to 15 years.

**Table 1:** Causes of conversion to THA after failed open reduction with internal fixation (ORIF).

Cause of failure	Number of cases
Secondary displacement	10 (32.3%)
Osteonecrosis	9 (29.1%)
Non-union/malunion	7 (22.6%)
Pseudarthrosis	2 (6.4%)
Early loosening	1 (3.2%)
Fracture	1 (3.2%)
Osteoarthritis	1 (3.2%)
Total	31 (100%)

Seven patients were deceased at the last follow-up after a mean period of 5.14 ± 3.13 years; the cause of death was not related to the hip surgery. No other patients were lost to follow-up. The mean time for conversion to THA was 2.03 ± 4.95 years, and the median was 1 year.

### THA characteristics

All patients had uncemented contemporary DMC (13 Quattro, Groupe Lepine, Genay, France, and 18 Avantage, Biomet, France). Twenty-nine patients had cemented femoral stem (CMK Institution, Groupe Lepine, Genay, France) and 2 patients had a Targos stem (Targos, Groupe Lepine, Genay, France). The acetabular cup ranged from 46 to 56 (median = 50). All femoral heads were in cobalt chrome; 4 had a size of 22.2 mm where the cup was smaller than 48 mm and the remaining had a size of 28 mm.

### Functional and radiological outcomes

No dislocation event was recorded post-operatively. No peri-prosthetic fracture occurred intraoperatively during stem implantation. No post-operative periprosthetic fracture was recorded at the last follow-up. One patient had a revision surgery following a septic loosening. This infection occurred in a 39-year-old male with no co-morbidities treated initially with cannulated screws for a FNF. Septic loosening was diagnosed 1 year following his primary THA. He was treated successfully with a two-stage revision THA. There were no cases of aseptic loosening. At the last follow-up, the mean mHHS was 92.9 ± 9.1. Twenty-two out of the 31 (71%) described their operated hip as a forgotten hip at the last follow-up.

A subgroup analysis was conducted to look for differences in the functional outcome between those who initially had intracapsular versus extracapsular FNF. The mean mHHS values were 85.2 ± 7.3 and 84.1 ± 9.1 (*p* = 0.8) for the intracapsular and extracapsular groups, respectively. Eight out of 12 (67%) and 16 (84.2%) out of 19 had a forgotten hip (*p* = 0.2), for the intracapsular and extracapsular groups, respectively.

Radiologically, there was no significant change in cup inclination, or cup radiolucency between the immediate post-operative period and the last follow-up. Sixteen stems (51.6%) were implanted in a neutral position, 13 (42%) in valgus (2.74 ± 1.72°) of which 1 with >5° in valgus, and 2 (6.4%) in varus (6.94 ± 2.02°) of which 2 with > 5° in varus.

## Discussion

Total hip arthroplasty after failed internal fixation is associated with a high complication rate [32, 33]. Several authors have conducted retrospective studies on THA conversion following failed intertrochanteric fractures using standard cups. Archibeck et al. [32] reported in 52 cases an early complication rate of 11.8% with a dislocation rate of 4.9% when using standard cementless acetabular component, along with cementless femoral fixation implants, and cemented stems in 50 cases. The rates of dislocation and periprosthetic fracture were higher when converting intertrochanteric extra-capsular fractures compared with converting femoral neck intra-capsular fractures. Using standard cementless and cemented acetabular cups, Tetsunaga et al. [8] reported a complication rate of 25%. Specifically, post-operative periprosthetic fractures and dislocations occurred at rates of 9.4% and 6.3%, respectively. The incidence of infection was 6.3%, and there was a single instance of stem perforation (3.1%). Zhang et al. [34] used standard cups in their THA and reported a complication rate as high as 47%. Intraoperative periprosthetic fractures and dislocations were observed in 32% and 16% of cases, respectively. In our study, no intraoperative complication or dislocation were encountered while only one patient had an infection presented as septic loosening.

Within this patient population, the risk of implant dislocation is a prominent concern, particularly in salvage procedures compared to primary surgeries [32]. This heightened incidence is attributed to factors such as implant misalignment, the presence of fragile scar tissue, and potential structural changes in the proximal femur, including the non-union of the greater trochanter [35]. The absence of dislocation is concordant with other articles reporting very low rates of instability [7, 26, 27]. In addition, we were interested in analyzing the femoral stem axis with regard to the femur axis. The healing of the initial fracture is likely to induce sclerotic bone which could be hard to ream and consequently favors the insertion of an undersized stem and/or malpositioning of the latter. Only two stems were found to be in varus while all the others were either in a neutral position or in slight valgus.

We did not encounter any intraoperative femoral fractures or post-operative femoral periprosthetic fractures. The rates of these complications vary in the literature, with intraoperative femoral fracture rates ranging from 0 to 32% and post-operative periprosthetic fracture rates between 0% and 5% [7, 26, 27, 32, 34, 36–39]. These reported rates were higher than in primary THA [40]. Many recommendations have been proposed in the literature in order to reduce the risk of intraoperative fracture. Angelini et al. [41] have advocated for dislocating the hip while keeping the internal fixation hardware intact. Archibeck et al. [32] proposed a preventive measure involving the use of prophylactic cerclage wiring over the most distal screw hole. Additionally, several studies have recommended bridging the final screw hole of the previous implant with a segment of at least 2 screw diameters [37, 42]. Based on our excellent outcomes, we do not recommend the systematic use of cemented or cementless long stems as suggested by some teams [39, 43]; these should be reserved for cases with severe bone loss and osteoporosis. We believe that the systematic use of a cemented femoral stem following a meticulous reaming of the femoral canal in this condition would avoid a peri-operative femoral fracture. Furthermore, the cement plug was cautiously placed under the last screw hole of the previous material.

While many studies reported high rates of complications in this population [8, 32, 34], Smith et al. underlined that patients treated with intramedullary nails had a higher number of complications compared to patients treated with other initial constructs: infection (6.2% vs. 2.6%), dislocation (8.1% vs. 4.5%), revision (8.4% vs. 4.3%), revision for infection (1.1% vs. 0.37%), and revision for dislocation (2.2% vs. 0.6%) [33]. In our study, we could not find outcome differences based on the initial type of internal fixation.

Only three studies reported outcomes related to the use of DMC in conversion to THA following failed PFF. Morice et al. found an advantage for DMC over standard cups with all their reported four dislocations occurring following the use of a standard cup [27]. The femoral stem positioning in their series was correctly aligned in the frontal plane in 83% of cases, 10% in varus position in 7% in valgus. Favreau et al. [26] highlighted the protective effect of DMC on implant dislocation for PFF fixation failure. With an overall high complication rate of 22%, there was no implant dislocation. Eight out of 40 (20%) included cases had a femoral stem malalignment at the last follow-up. Boulat et al. [7] reported a rate of 21% of complications (6 intraoperative femur fractures, 1 post-operative dislocation, and 1 post-operative infection in a total of 33 patients) only in patients who initially underwent intramedullary nailing and only for intertrochanteric fractures. The only dislocation event they reported was after a fall and was considered post-traumatic.

Our study reported excellent functional outcomes post-operatively with no dislocation, with a mean mHHS of 92.9 ± 9.1. We demonstrated that 71% of our population described their hip as a forgotten hip at the last follow-up, and regained their initial pre-fracture level of activity. This is in concordance with many published results reporting the use of DMC in high-risk patients [7, 26, 27].

The present study has some limitations. The moderate sample size could have an impact on the conclusions; nevertheless, it reflects the rarity of such conversion surgery. However, our reported outcomes of DMC used in high-risk patients [13, 17–19, 23] are in line with those of this study. The retrospective study design with no comparator could limit the degree of evidence. However, this is comparable in terms of sample size and study design to other previously published studies in the literature [7, 40]. Though some surgical technical aspects could differ among studies, in our study the conversion surgery was performed by the same senior surgeon using a systematic surgical technique with contemporary DMC implants.

## Conclusions

The use of DMC in THA has a protective effect against instability when used after failure of internal fixation for proximal femoral fractures. Our results demonstrated no dislocation event or peri-operative femoral fracture. The findings highly support the use of contemporary DMC in this high-risk population to reduce peri- and post-operative complications.

## Conflict of interest

The authors declare no competing interests with regard to this article.

## Funding

This research did not receive any specific funding.

## Ethical approval

Ethical approval was obtained from our institution’s Ethical Committee prior to its commencement.

## Informed consent

Obtained for the figures. The Ethic committee of the institution waved this condition since the study was retrospective based on the charts of the patients.

## Authors contributions

Concept of the study: KY and CA. Data extraction: JO, JM, JD. Data analysis: KY. First draft: All authors. Review and approval: All authors.
